# Changes in autofluorescence based organoid model of muscle invasive urinary bladder cancer

**DOI:** 10.1364/BOE.7.001193

**Published:** 2016-03-07

**Authors:** Scott Palmer, Karina Litvinova, Andrey Dunaev, Stewart Fleming, David McGloin, Ghulam Nabi

**Affiliations:** 1 Division of Imaging and Technology, University of Dundee, Ninewells Hospital and Medical School, James Arrott Drive, Dundee, DD1 9SY, UK; 2 Optoelectronics and Biomedical Photonics Group, Aston Institute of Photonic Technologies, Aston University, Aston Triangle, Birmingham, B4 7ET, UK; 3 Biomedical Photonics Instrumentation Group, Scientific-Educational Centre of “Biomedical Engineering,” State University – Education-Science-Production Complex, Oryol, 302020, Russia; 4 Medical Research Institute, University of Dundee, Ninewells Hospital and Medical School, James Arrott Drive, Dundee, DD1 9SY, UK; 5 Division of Electronic Engineering and Physics, Ewing Building, University of Dundee, Nethergate, Dundee, DD14HN, UK

**Keywords:** **(**000.1430) Biology and medicine, (170.6510) Spectroscopy, tissue diagnostics

## Abstract

Muscle invasive urinary bladder cancer is one of the most lethal cancers and its detection at the time of transurethral resection remains limited and diagnostic methods are urgently needed. We have developed a muscle invasive transitional cell carcinoma (TCC) model of the bladder using porcine bladder scaffold and the human bladder cancer cell line 5637. The progression of implanted cancer cells to muscle invasion can be monitored by measuring changes in the spectrum of endogenous fluorophores such as reduced nicotinamide dinucleotide (NADH) and flavins. We believe this could act as a useful tool for the study of fluorescence dynamics of developing muscle invasive bladder cancer in patients.

Published by The Optical Society under the terms of the Creative Commons Attribution 4.0 License. Further distribution of this work must maintain attribution to the author(s) and the published article’s title, journal citation, and DOI.

## 1. Introduction

Carcinoma of the urinary bladder is one of the most common urological cancers (4^th^ most frequent malignant disease in men and 7^th^ most frequent in women). In 75–85% of these the cancer does not infiltrate into deeper muscle, i.e. it is carcinoma in situ (CIS), stage Ta/T1 (non-invasive) on first diagnosis; 50–70% of these patients presenting with superficial (non-muscle invasive) stages have one or several recurrences after the initial treatment, and in ≈15% there is disease progression [1]. Sensitivity and specificity of current cystoscopy and urinary biomarkers including cytology are far from satisfactory, leading to use of invasive cystoscopy as a follow-up investigation. The high rates of recurrence in non-muscle invasive urinary bladder cancer and its progression make it one of the most expensive cancers to treat on a per patient basis [2–4]. One of the major developments in increased sensitivity diagnosis of bladder cancer was the advent of photodynamic diagnosis (PDD) using photosensitizers (PS), including 5-aminolaevulinic acid. Unfortunately, PS agents are also retained and converted preferentially by inflamed or infected tissue, reducing diagnostic specificity. Therefore, PDD causes many more false positives than white light cystoscopy (WLC), leading to increased biopsy turnover and increased patient morbidity [5,6]. It is clear that something must be done to balance this.

In recent years, endogenous fluorescence spectroscopy has been gaining attention as a means to diagnose and monitor bladder cancer with improved sensitivity without compromising on specificity. The technique is based upon the excitation of particular endogenous fluorophores, including collagen, elastin and reduced Nicotinamide Dinucleotide (NADH) [7], which can give a wealth of structural and metabolic information about tissue [8]. Many recent studies have demonstrated the diagnostic worth of endogenous fluorescence spectroscopy in several cancers [9], including bladder cancer [10], however this information is often conflicting. Endogenous fluorescence spectroscopy has demonstrated the ability to discriminate and quantify tissue and cellular levels of fluorophores [11], and the calculation of ratios including the optical redox ratio [12–14] of tissues can provide complex information on how cellular systems progress. Clinical diagnostic implementation of endogenous fluorescence spectroscopy relies on consensus being reached regarding diagnostically relevant fluorophores and their dynamics in healthy and cancerous tissue. Furthermore, systems allowing us to monitor fluorescence changes in tissue as it progresses from healthy to CIS to non-muscle invasive bladder cancer (NMIBC) and finally to invasive cancer could inform grading and staging and help to improve disease monitoring and follow-up.

We have developed an *in vitro* organoid model of invasive bladder cancer following previous work by Southgate et al. [15] to allow us to monitor fluorescence dynamics in the lab using a minimally invasive spectroscopic probe. Organoids are becoming widely used tissue mimics in labs worldwide as they allow the exploration of minute cell and tissue details in close to lifelike conditions. Organoids have previously been developed for many tissue types, ranging from colon [16] to prostate [17], and utilise tissue from many animal types, including rat, pig and human explants. Previous work in the field of bladder organoids has established spheroids [18], reliable, small scale mucosal models from rats [19] and full tissue models from porcine bladder, mostly concerned with tissue mimics for drug development [20] and disease recurrence [21] and invasion assays. To our knowledge, there are no existing studies employing bladder cancer organoids to study tissue autofluorescence dynamics, therefore ours represents the first of its kind. We hypothesised that tumour organoids developed in this way would express different levels of fluorophores from control tissue across the study period – in particular we anticipated increased cellular fluorophores such as NADH and flavins as tumour cells adhered and formed an epithelial layer on tissue, potentially alongside reduced collagen and elastin levels as tumour cells remodelled the surrounding matrix to promote growth and invasion.

The fluorescence data obtained over a 21 day study period display definitive changes in several fluorophore ratios, suggestive of progressive cancer cell epithelialisation of tissue and destruction of structural proteins to facilitate tissue invasion.

## 2. Materials and methods

### 2.1 Porcine bladder scaffold

**Fig. 1:**
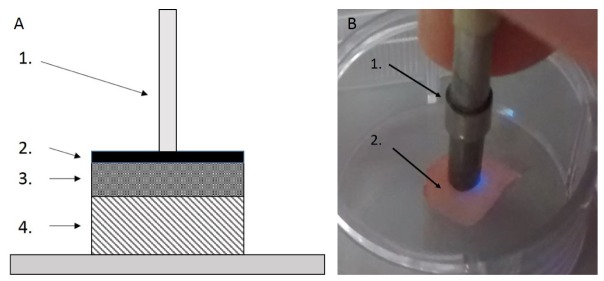
Setup of optical probe in relation to tissue scaffold showing (a) a schematic cross-section of tissue and optical probe (1 = optical probe, 2 = mucosal layer, 3 = connective tissue and 4 = muscle layer), and (b) an image of the optical probe in contact with the mucosal surface of tissue (1 = optical probe, 2 = mucosal surface of tissue scaffold).

Porcine bladders from freshly slaughtered pigs were purchased from Medical Meat Supplies Ltd. The tissue was halved to reveal the urothelial surface of the bladder. From one half of the bladder, a 20 cm^2^ (4cm x 5cm) section was cut with a scalpel. From this, the mucosa and a small section of muscle were cut away from the remaining muscle layer, to give a tissue scaffold with a remaining thickness of roughly 2-3mm. The mucosa/muscle layer was kept and the remaining muscle was discarded. Following this, the tissue scaffold was cut into 20 identical sections of 1cm^2^, washed x 3 in Dulbecco’s Phosphate Buffered Saline (DPBS) (Sigma Aldrich) to remove leftover urine or contaminants, sterilised in 10,000 Units Penicillin- 10mg Streptomycin solution (Sigma Aldrich) and transferred to 0.25% Trypsin – ethylenediaminetetraacetic acid (EDTA) solution (Sigma Aldrich) and incubated for 15 minutes to disrupt urothelium, following which they were again washed in DPBS x 3 and placed in individual wells of a Corning Costar 12 well plate (Sigma Aldrich). A cross section of the tissue scaffold (1a) and an image of the optical probe in contact with the tissue (1b) are included in Fig. 1

.

### 2.2 Cell culture

Bladder cancer cell line “5637” was purchased from American Type Culture Collection and grown for several passages in Dulbecco’s Modified Eagle Medium (DMEM) (Sigma Aldrich) supplemented with 10% Fetal Bovine Serum (FBS) (Sigma Aldrich) and 1% Penicillin-Streptomycin (Sigma Aldrich) in 75cm^2^ cell culture flasks (Corning). At 70% confluence, cells from one flask were detached by conventional trypsinisation, resuspended in DMEM, split into ten 15ml Falcon tubes (BD Biosciences) and spun at 1500RPM for 10 minutes to form pellets. Pellets were applied to ten of twenty bladder scaffolds, leaving ten scaffolds as a control. The scaffolds and cell pellets were resuspended in 1ml DMEM and incubated overnight.

### 2.3 Fluorescence spectroscopy

**Fig. 2:**
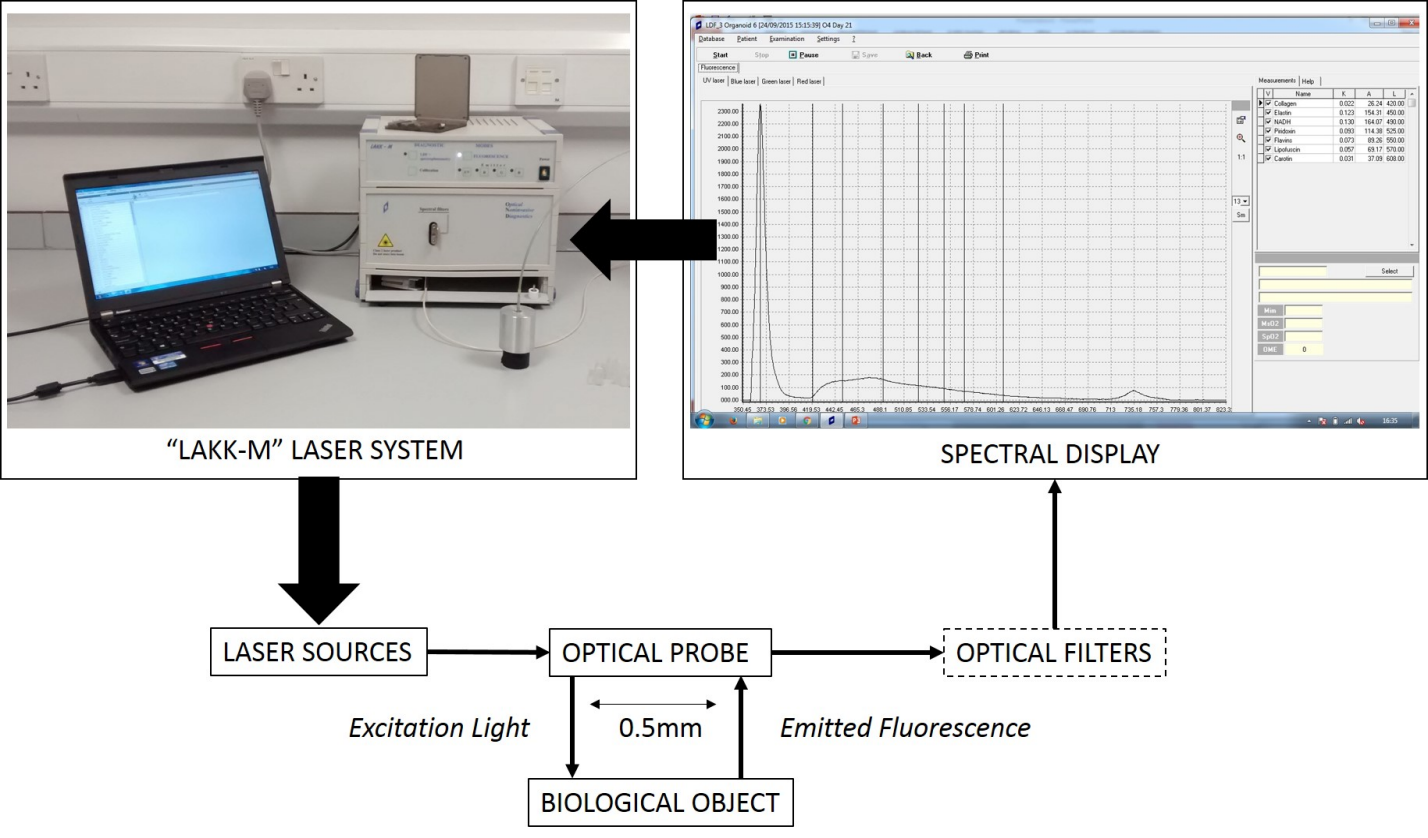
Experimental setup of the fluorescence channel of the “LAKK-M” multi-functional system (top left). The system is controlled through the attached laptop (top left) to activate laser sources (UV; blue; green and red). Excitation light from the chosen source is directed in a source fibre through an optical probe to the biological object (in this case, organoid). Emitted fluorescence is transmitted through the detection fibre of the optical fibre, through optical filters to the CCD matrix, then finally displayed as a complex fluorescence spectrum on the custom software (top right). Spectroscopy interface displays a peak of back-scattered laser light (first peak) and a complex fluorescence spectrum (second peak) alongside recorded fluorescence intensity values for each fluorophore.

Fluorescence spectroscopy was performed using a multi-functional laser based non-invasive diagnostic system “LAKK-M” (SPE“LAZMA” Ltd, Russia). The “LAKK-M” system consists of a central functional unit containing the excitation sources for fluorescence diagnosis, laser Doppler flowmetry and tissue reflectance oximetry (these two methods not used in this study) and emission detector. These are coupled to a fibre optic probe of 1mm diameter with a spacing of 0.5mm between the source and detector. The system was initiated 30 minutes prior to study then transferred to the cell culture lab. Measurements were performed at the same time on all samples in the afternoon of each day on days 0, 1, 4, 7, 11, 14, 18 and 21 of study by gently touching, without pressing down, the probe of the “LAKK-M” to the exact centre of the mucosal (upward facing) surface of tissue each time. On day 0, tissue was measured prior to seeding of cancer cells to ensure all tissues displayed similar fluorescence. Fluorescence measurements were performed in the dark to prevent interference from background noise. The “LAKK-M” system excites and measures fluorescence across 4 excitation channels: UV (365nm); blue (450nm); green (532nm); red (633nm) – samples were studied sequentially for each wavelength. Amplitudes of fluorescence excited at each wavelength were detected in the detector channel of the probe, converted to electronic signals via a charge coupled device (CCD) -matrix and recorded as complex spectra using an in-built spectrometer coupled to the detector probe. The spectrometer measures intensity across a wavelength range of 350-823nm at 0.2nm increments. Spectra recorded from tissue was visualised on an accompanying laptop using custom-made software (LDF 3 v3.1.1.403, SPE “LAZMA” Ltd, Russia). Full experimental setup of the fluorescence channel of the LAKK-M system is presented in Fig. 2

and more information about the technical specifications of the LAKK-M system can be found in previous work by Dunaev et al [22].

### 2.4 Confirmation of invasion

**Fig. 3:**
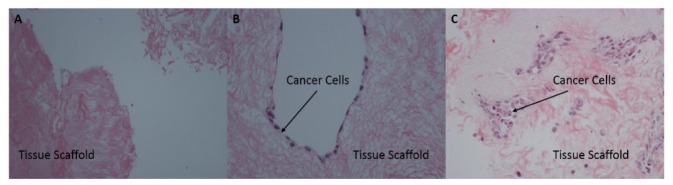
H & E stained slides showing control (a), cancer cell attachment (b) and cancer cell invasion (c).

A pilot study was run using the steps described, but for two and twelve days, after which the samples were fixed in formalin and submitted to the Tayside Tissue Bank, Ninewells Hospital, Dundee, for sectioning and staining of tissue slides with Haematoxylin and Eosin. Attachment of cancer cells to scaffold in organoids by day two, and T1 transitional cell carcinoma phenotype by day 12, were confirmed by an experienced pathologist. Slides were imaged at 200x magnification using a Nikon Eclipse E600 microscope. H & E stained slides of control (3a), attachment (3b) and invasion (3c) are included below (Fig. 3

):

### 2.5 Data analysis

Data was analysed using custom made analysis software LDF 3 v3.1.1.403 (SPE “LAZMA” Ltd). The software calculates and records both the amplitude of fluorescence (Af) and coefficient of fluorescent contrast (Kf) at distinct pre-set wavelengths in accordance with previously published literature [22–24]. The fluorophores of interest and their excitation and emission values were as follows (Table 1

**Table 1:** The most common endogenous fluorophores

**Fluorophore**	**Excitation (nm)**	**Emission (nm)**
Collagen	365	420
Elastin	365	450
NADH	365	490
Flavins	365	550
Flavins Blue	450	510
Lipofuscin	532	570
Carotene	532	608
Porphyrins	633	710

):

Optical redox ratio (a measure of metabolic function) was calculated for each sample on each day as NADH (ex365 em490)/Flavin (ex365 em550) and as NADH (ex365 em490)/Flavin (ex450 em510). Additionally, the following ratios were also calculated: Elastin/NADH; NADH/Collagen and Elastin/Flavin. Average and ratio values were compared by plotting mean + SEM (Standard Error of the Mean) for control versus organoid over the 21 days of study. One control sample was removed from study on day 4 owing to fungal contamination. Two further control samples were removed on day 18 due to fungal contamination. Collected data were analysed and plotted as graphs using Origin Pro 8 (OriginLab). Data were compared and assessed for statistically significant differences at each time point using AOV (ANOVA) test in R Studio (leverage of residuals and Q-Q plots were assessed to ensure applicability of analysis) for p value with p < 0.05 considered statistically significant. Raw data from fluorescence analysis and statistical analysis have been stored on a secure computer for any potential future access.

## 3. Results

### 3.1 Optical redox ratio

**Fig. 4:**
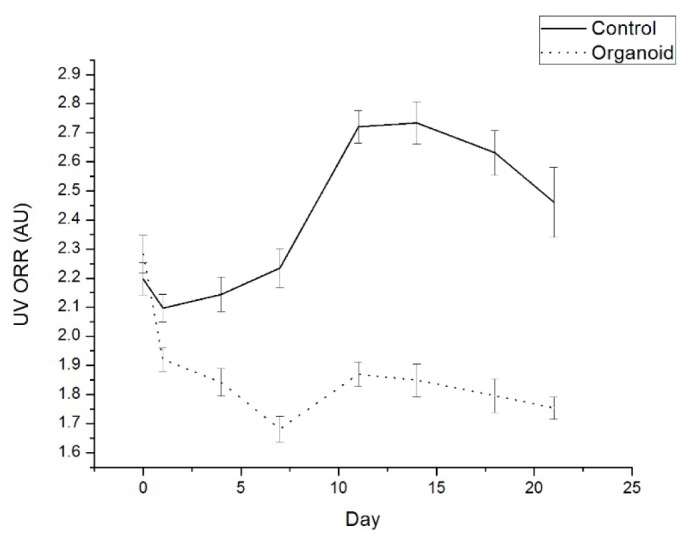
Average ORR values across experimental period for control (solid line) and organoid (dotted line). Error bars represent standard error of the mean.

Optical redox ratio (ORR) values (measured as NADH (ex365em490)/flavins (ex365em550)) show a sharp decrease in organoids, from equitable levels to control at day 0 to progressive decreases across day 1-21 (Fig. 4

). Data at day 0, before addition of cancer cells, were found to not be statistically significantly different (p = 0.334), however data on each subsequent day were found to be statistically significantly different (p < 0.05). Day to day variations in environment and instrumentation are accounted for by control. ORR calculated using flavins measured under blue excitation (ex450em510) showed no significant difference over the course of the experiment (p > 0.05). This could be a result of slight movement of the optical probe when switching from UV to blue excitation sources.

### 3.2 Elastin relative to metabolic cofactors

**Fig. 5:**
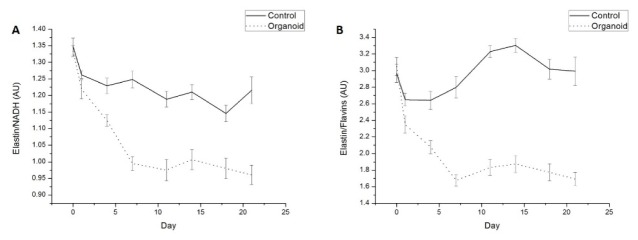
Average ratios of Elastin/NADH (a) and Elastin/Flavins (b) across experimental period in control (solid line) and organoid samples (dotted line). Error bars represent standard error of the mean.

Taking the ratio of elastin fluorescence (ex365em450) against that from both the metabolic cofactors NADH (ex365em490) (5a) and flavins (ex365em550) (5b) also shows significant decreases in the relative elastin levels compared to these fluorophores in organoids across the 21 day period (Fig. 5

). For elastin/NADH measurements, data at day 0 and day 1 were not statistically significant (p = 0.668 and p = 0.146, respectively), however differences in data from each subsequent day was statistically significant (p < 0.05). Data from elastin/flavin measurements on day 0 were not statistically significant (p = 0.598), however differences in data were statistically significant on each subsequent day (p < 0.05). Data from NADH/collagen showed no consistent statistically significant changes across the experimental period. Furthermore, data for this ratio on day 0 prior to addition of cancer cells was statistically significant (p = 0.0182). This is possibly due to very low relative fluorescence of collagen recorded at ex365em420 making it susceptible to background noise interference.

All data created during this research are openly available from the University of Dundee data repository at http://dx.doi.org/10.15132/10000108

## 4. Discussion

From the data presented in this paper, we can clearly see patterns of fluorescence emerge as tissue scaffold is adhered to and invaded by the cancer cells. We have assessed fluorescence from distinct endogenous tissue fluorophores using the “LAKK-M” diagnostic system and based on analysis techniques previously followed by Dunaev et al. [22] and Akbar et al. [23]. Previous studies generating bladder organoids from porcine bladders have demonstrated viability of cells within tissue scaffolds up to 3 weeks after induction [25]. From this we concluded that 3 weeks would be a suitable time frame for our fluorescence study. Our work has shown that the UV spectrum of organoids changes relative to control across the experimental period. To investigate why this occurred, we have analysed the dynamics of several endogenous fluorophores across the study period. The study of individual fluorophores is not well suited to this type of spectroscopy, as day to day variations in both laser power and tissue condition can affect amplitudes of fluorescence. We therefore found no consistent patterns of fluorescence from individual fluorophores. Instead, we looked at ratios of particular fluorophores relative to one another. The measurement of ratios, as used previously by Georgakoudi [7], Ostrander [13] and ourselves [11], among others, provides a much more robust measurement of fluorescence dynamics. In particular, we focussed on the metabolic cofactors NADH and flavins, and the structural protein elastin, all known to fluoresce under UV excitation. As the source of NADH and flavins is primarily cellular, our conclusion is that the progressive decrease in ratios of elastin/NADH and elastin/flavins may reflect adherence, epithelialisation and invasion of tissue by cancer cells. Furthermore, it may also directly reflect the progressive destruction of the structural protein elastin in the surrounding connective tissue of the mucosa, lamina propria and muscular layers as cancer cells attach and begin to secrete digestive enzymes such as matrix metalloproteinases to promote invasion. We know from previous studies that the urothelium (2-3 cells thick) contributes significantly to the NADH and flavin signals present in bladder tissue. In optical biopsies obtained by multi-photon microscopy, intense autofluorescence can be observed on the top layers of urothelium with strong second harmonic generation (SHG) signal from collagen observed below [26]. We consider that the main fluorophores contributing to organoid fluorescence may be NADH and flavins from the new tumour urothelium, while control fluorescence may contain a higher contribution from the underlying collagen and elastin matrix of the lamina propria and extra-cellular matrix (ECM). Previous studies have suggested increased optical redox ratios (increased NADH relative to flavins) in cancer cells and tumours compared to healthy controls [11]. We see the opposite of this – a reduced optical redox ratio in the tumour organoid. This could be a result of changes in structural proteins, rather than changes in metabolism. As the excitation and emission profiles of elastin overlap somewhat with that of NADH, the complex fluorescence spectrum arising from UV excited tissue likely contains an NADH signal at 490nm with underlying contribution from elastin. The epithelialisation of organoids results in increased cellular fluorophores, therefore the reduced ORR seen in organoids may be a function of the increased Flavin to elastin ratio we observe rather than a reduced NADH – flavin couple. We suggest from this research that studying the relative changes in elastin and collagen compared to NADH and flavins may serve as a useful tool for monitoring bladder tumour development. In this work, we have demonstrated a method for studying the dynamic changes in fluorophore levels during colonisation and invasion of bladder tissue by tumour cells. That cells were still viable by day 21 of the study suggests this model could be further used for evaluation of fluorescent responses to drug and treatment regimens such as Mitomycin C, akin to microscopy work done by Walsh et al. for breast cancer [27]. Moving forward, it would also be interesting to study the fluorescence dynamics of colonisation of scaffolds by cell lines representing different grades of urinary bladder cancer. We could then identify, using fluorescence spectroscopy, critical time points where fluorescence differs, giving a clinical insight into spectral cues for malignancy and invasion in bladder cancer. The application of multi-photon and second harmonic generation (SHG) imaging to the study of these organoids may allow us to develop a better understanding of cellular and structural behaviour. Finally, information gleaned on the fluorescence dynamics of developing tumour *in vitro* may allow us to inform and develop currently used methodologies of computer simulation and modelling of the disease [28].
